# Modulation of Adult Hippocampal Neurogenesis by Early-Life Environmental Challenges Triggering Immune Activation

**DOI:** 10.1155/2014/194396

**Published:** 2014-05-07

**Authors:** Ksenia Musaelyan, Martin Egeland, Cathy Fernandes, Carmine M. Pariante, Patricia A. Zunszain, Sandrine Thuret

**Affiliations:** ^1^Centre for the Cellular Basis of Behaviour, Institute of Psychiatry, King's College London, The James Black Centre, 125 Coldharbour Lane, London SE5 9NU, UK; ^2^Department of Psychological Medicine, Institute of Psychiatry, King's College London, The James Black Centre, 125 Coldharbour Lane, London SE5 9NU, UK; ^3^MRC Social, Genetic & Developmental Psychiatry Centre, Institute of Psychiatry, King's College London, 16 De Crespigny Park, London SE5 8AF, UK

## Abstract

The immune system plays an important role in the communication between the human body and the environment, in early development as well as in adulthood. Per se, research has shown that factors such as maternal stress and nutrition as well as maternal infections can activate the immune system in the infant. A rising number of research studies have shown that activation of the immune system in early life can augment the risk of some psychiatric disorders in adulthood, such as schizophrenia and depression. The mechanisms of such a developmental programming effect are unknown; however some preliminary evidence is emerging in the literature, which suggests that adult hippocampal neurogenesis may be involved. A growing number of studies have shown that pre- and postnatal exposure to an inflammatory stimulus can modulate the number of proliferating and differentiating neural progenitors in the adult hippocampus, and this can have an effect on behaviours of relevance to psychiatric disorders. This review provides a summary of these studies and highlights the evidence supporting a neurogenic hypothesis of immune developmental programming.

## 1. Introduction

Adult hippocampal neurogenesis is a complex process which includes continuous renewal of the hippocampal stem cell pool and generation of new neurons in the dentate gyrus (DG) of the hippocampus during adult life [1]. As neurogenesis is considered to be an important contributor to the neuroplastic ability of the hippocampus, it is suggested that it can be influenced by multiple environmental factors. The immune system plays a key role in the communication between the body and the environment [2]. Therefore investigating the relationship between the immune system and hippocampal neurogenesis can provide an important insight into the impact of environmental factors on a neurogenic niche, a region where neurogenesis occurs in a regulatory microenvironment. Recent research indicates that adult hippocampal neurogenesis is indeed extensively affected by elements of the immune response such as activated microglia and cytokine release. Immune activation has been shown to play an important role in many neuropsychiatric diseases such as Alzheimer's disease, schizophrenia, autism, and depression [3]. Importantly, a growing number of studies report that immune activation* in utero* can augment the risk of these disorders in adulthood. Accordingly, maternal infections have been associated with increased risk of schizophrenia and autism [4, 5]. In depression, early-life factors which are known to increase the risk of the disorder in adulthood, such as maternal anxiety and perinatal depression as well as traumatic postnatal life events, are associated with changes in the immune system [3, 6, 7]. This evidence suggests that environmental factors in early-life predispose individuals to the development of psychopathological conditions in adulthood, potentially via the activation of the immune system. The mechanism of such developmental programming is unknown; however some preliminary evidence is emerging in the literature which suggests that adult hippocampal neurogenesis may be involved. The neurogenic hypothesis of immune developmental programming is still in its early days and this review will summarize the emerging supporting evidence available to date.

## 2. The Effect of Inflammation on Hippocampal Neurogenesis in Adult Life

The relationship between inflammatory factors and adult hippocampal neurogenesis has been a focus of multiple studies during recent years. Most of the knowledge in this area comes from studies which used lipopolysaccharide (LPS) or cytokine administration to model the inflammatory state. LPS is a bacterial endotoxin which is commonly used to reproduce an immune response where its systemic administration induces a behavioural phenotype known as “sickness behavior,” through an increase of the levels of proinflammatory cytokines in peripheral blood as well as in the brain [8]. The central effect of LPS can be attributed to the activation of microglia, which is known to release cytokines in the activated state. In 2003, Ekdahl and coworkers [9] demonstrated that direct intracortical administration of LPS dramatically reduced the survival of newly generated neurons in the adult hippocampus, while Monje and coworkers found the same effect of peripheral LPS administration [10]. Other studies supported the role of individual cytokines in this process, demonstrating that overexpression of interleukin 6 (IL-6) and interleukin 1 beta (IL-1*β*) in the brain as well as systemic administration of tumor necrosis factor alpha (TNF-*α*) can reduce cell proliferation and neuronal differentiation in the adult hippocampus, stimulating more progenitor cells to differentiate into astrocytes [11–13]. An* in vitro* study which employed human hippocampal stem cell line confirmed that this effect also occurs in human hippocampal cells [14]. Moreover it appears that not only the production, but also the function of new neurons is affected by inflammation. Accordingly, Belarbi and coworkers showed that chronic exposure to LPS decreased the recruitment of new neurons into hippocampal networks following training in a spatial exploration task [15]. This further suggests that neurogenesis might be responsible for the cognitive deficits seen in infectious diseases accompanied by inflammation. Currently, microglial activation and cytokine release are also suggested to be an underlying mechanism of neurogenic decline in noninfectious conditions such as Alzheimer's disease and normal aging [16].

## 3. Programming Effects of Early-Life Immune Activation

### 3.1. Early-Life Immune Activation in Humans: Consequences for Adult Behaviour and Psychopathology

Studies have shown that inflammation plays a specific neuroregulatory role not only in adult life and aging, but also in early development. Thus clinical evidence suggests that prenatal exposure to maternal infections predisposes an individual to the development of neuropsychiatric diseases such as schizophrenia and autism [5]. However the causality of immune activation extends beyond infectious conditions. It has been shown that other environmental factors apart from exposure to infectious agents can activate the immune system in mother and child. Nutritional status of mother-infant dyad can have a profound effect on the developing immune system. While maternal nutrition during pregnancy insures sufficient availability and transfer of immune factors to the foetus and can influence the development of the immune system, postnatal nutrition has an additional function of introducing the infant's immune system to food antigens and subsequent development of optimal immune tolerance of the gut. Impaired immune tolerance can lead to gut inflammatory disease and food allergies, conditions which lead to chronic activation of the immune system [17]. Moreover, food is one of the most important sources of environmental contaminants, toxic compounds created by industrial activity. Exposure to these xenobiotics can lead to developmental immunotoxicity (reviewed in [6]). Importantly, psychosocial factors can also affect immune system in the early-life. Thus maternal stress and anxiety have been shown to affect immune communication between mother and foetus which in turn affects the postnatal immunological status of the child. As such, maternal anxiety has been associated with reduced adaptive immunity in infants [18], while prenatal exposure to stress has been linked to increased risk of infectious disease-related hospitalisation in childhood [19]. Similarly for postnatal stress, Danese and colleagues described a positive association between childhood maltreatment and an increase in the inflammatory marker C-reactive protein in adulthood [7].

### 3.2. Early-Life Immune Activation in Animal Models: Consequences for Adult Immune and Stress Response Regulation

While human data in this field are still quite limited, animal studies provide abundant evidence demonstrating the link between perinatal immune activation and consequences for the immune system and stress susceptibility in adulthood. These studies, in line with the inflammation and neurogenesis studies described above, also use LPS to activate the immune system; however* E. coli* infection was employed by some research groups to stimulate immune response [20–22]. Most of the studies show that immune stimulation over the first few days of life leads to changes in the cytokine production in the brain in response to LPS exposure in adulthood. Thus Bilbo and colleagues showed that neonatal* E. coli* infection at PND 4 led to an increase in IL-1*β* protein shortly after (1.5 hrs) adult LPS exposure. Interestingly this effect was specific for the hippocampus and parietal cortex [21], while hypothalamic IL-1*β* protein content was decreased in the same conditions [22]. These changes also correlated with hippocampal function, as neonatally exposed rats displayed memory impairment in the contextual memory task following LPS injection in adulthood [22]. Furthermore, neonatally infected rats had persistently elevated levels of microglial activation markers such as major histocompatibility complex II (MHCII) and Iba-1 in the hippocampus in adulthood [20, 22, 23]. This increase may suggest that hippocampal microglia remained in a “primed” state during adulthood, characterised by an exaggerated proinflammatory response when exposed to the immune challenge [24]. Interestingly, the direction of observed change in cytokine content might be time- and dose-specific. This is exemplified in a study by Kohman et al. who observed an opposing decrease in hippocampal IL-1*β* gene expression looking specifically at expression in 4 hours (rather than 1.5 hours) after LPS injection in an otherwise similar study design [25]. While this decrease can be a compensatory response to an elevation of the protein content observed at 1.5 hrs after the injection [21], it is also possible that the difference in the direction of the response is due to the dose difference between the studies, as the dose of LPS used by Kohman et al. to challenge immune system in adulthood is 10 times higher than that described in the previous studies [20–22]. Nonetheless, these studies indicate that neonatal infection can modulate adult immune response in the hippocampus. Interestingly, none of these studies found change in peripheral cytokine levels in neonatally exposed animals. However, a study which exposed rat pups to LPS at a later time point, PND 14, described an attenuated elevation of blood cytokines in response to LPS challenge in adulthood [26]. Such discrepancy demonstrates that the timing of postnatal exposure can significantly influence the type of response observed in adulthood. Indeed it has been shown that some of the proteins involved in immune response to antigens such as LPS-binding protein (LBP) are not expressed during the first week of life [27]. Interestingly LBP expression peaks at PND 14, which might explain the more robust effects observed in Ellis et al.'s study described previously.

Early-life inflammatory challenges have also been shown to cause behavioural changes in adulthood. This is exemplified by how the stress response in adulthood has been shown to be modulated by early-life LPS exposure with many studies showing increased fear and anxiety-like behaviour and decreased exploratory behaviour in relevant tasks such as elevated plus maze and open field/hide box in response to restraint stress [28–30]. The underlying mechanism of this outcome is suggested to be the programming effects of early-life LPS exposure on hypothalamo-pituitary-adrenal (HPA) axis regulation. This notion is supported by observed changes in endocrine factors on each level of the HPA axis. More specifically, increased baseline corticotropin-releasing hormone (CRH) expression in the paraventricular nucleus of the hypothalamus [31], increased plasma concentrations of adrenocorticotrophic hormone (ACTH), increased and/or prolonged adrenal corticosterone release in response to stress in neonatally LPS-exposed animals [31–34], and decreased glucocorticoid receptor (GR) density in the hypothalamus, hippocampus, and frontal cortex of adult animals, where GR is thought to play a key role in negative feedback regulation of the HPA axis [31].

Further aspects of behaviour such as reproductive function and memory have also been shown to be affected. Thus Wu et al. showed that female rats exposed to LPS in early-life displayed a significant delay in puberty and disruption of oestrous cycle which persisted into adulthood [35]. Moreover it has been demonstrated that neonatal LPS exposure leads to a decrease in inflammatory stress resilience of the female reproductive system by long-term sensitization of gonadotropin releasing hormone (GnRH) regulator, a central controller of reproduction. This effect was reflected by increased suppression of luteinising hormone frequency in response to adult LPS exposure [36]. In male animals, early-life LPS exposure also has been shown to affect reproductive function. Accordingly, Walker et al. demonstrated that neonatally exposed male animals show reduced sexual activity following stress, indicated by reduced amount of mounts and ejaculation when presented with a female [37].

Learning- and memory-related behaviours have also been reported to be affected by prenatal or neonatal inflammatory agent exposure. Specifically, Kohman et al. showed that neonatally exposed rats show associative learning and memory impairment in active avoidance conditioning task [25], while a similar effect was observed in this task for adult offspring of mice subjected to poly I : C injections during pregnancy [38].

Thus, research demonstrates that early-life exposure to inflammatory stimuli modulates multiple aspects of development from the immune system to the stress response and, furthermore, that some of these effects are specific for the hippocampus. Importantly both immune system activation and chronic stress are implicated in psychopathological conditions such as anxiety and depression. Therefore, investigating the mechanisms through which early-life immune activation exerts such a long-lasting effect on these systems might shed light on the means by which early-life environmental insults predispose individuals to the development of psychopathology in adult life.

As some of the changes found to be affected by inflammation early in development were specific for the hippocampus, it follows that the hippocampus would be a first target in search of the underlying mechanism. Indeed, the hippocampus is an interesting brain area as it has been recently confirmed that it is the only area of the human brain where neurogenesis occurs throughout adult life [39]. Hippocampal neurogenesis has been extensively implicated in mood disorders and in the mechanism of antidepressant action [40, 41]. Therefore it appears that modulation of neurogenesis by early-life immune activation might play a role in the long lasting effects of the perinatal inflammatory insult. However, the study of early-life inflammatory modulation of adult hippocampal neurogenesis has only recently attracted researchers' attention. The next section will describe the emerging studies published to date which introduce this new important field of neurogenesis research and suggest directions for future investigation.

## 4. Perinatal Immune Activation and Adult Hippocampal Neurogenesis

### 4.1. Methodological Considerations in the Study of the Effect of Perinatal Immune Activation on Adult Hippocampal Neurogenesis

Studies investigating the effect of early-life inflammation on neurogenesis have employed similar models to activate the immune response as those dedicated to the effects of inflammatory challenge on immune response or behaviour in adulthood. LPS injection is a common way to activate immune response, as LPS is recognised by toll-like receptor 4 (TLR4) on antigen-presenting cells and effectively models bacterial infection. In the case of prenatal exposure, maternal antigen-presenting cells may interact with the placenta, capable of producing its own cytokines, which then enter foetal bloodstream [24]. A viral mimetic poly I : C also has been used in some of the studies. Poly I : C stimulates TLR3 receptor which recognises viral antigens. As it has been shown that maternal influenza increases the risk of schizophrenia in the offspring [4], poly I : C also appears to be a valid model to study long-term effects of perinatal inflammation.

Timing of the inflammatory exposure is another crucial point in the study design, as some reports suggest that different phases of pregnancy represent unique vulnerability windows to environmental inputs [42]. Thus the rodent prenatal period can be approximately correlated with human pregnancy as gestation days 1–9 (GD 1–9) being representative of first trimester of human pregnancy, GD 10–19 as a second trimester, and GD 19-20 to postnatal day (PND) 7 as corresponding to the third trimester of pregnancy [43]. It is important to note however that an exact comparison is not possible as the course of rodent and human pregnancy and brain development differ significantly; therefore some discrepancies exist between research groups in the interpretation of gestational timing.

The timing of the assessment of hippocampal neurogenesis is also an important factor to consider in the study design. Thus neurogenesis assessed before PND 21, when offspring is usually weaned from the mother, will represent the state of juvenile development, the period between PND 21 and 50, a period when rodents reach reproductive maturity [37], as adolescence and early adulthood, while PND 60 onwards is generally agreed to be representative of adulthood [44].

Studies also differ in which aspects of the neurogenesis were assessed and the methodological approaches used to characterise them. For example, cell proliferation assay, usually assessed using bromodeoxyuridine (BrdU) incorporation or KI67 immunohistochemistry, shows the rate of proliferation of the neural progenitors within the dentate gyrus. This is an important marker of the renewal of the pool of neural progenitors, which is thought to be crucial to maintain neurogenic capacity of the hippocampus throughout life [45]. However, this assay does not provide any information on the subsequent fate of the new cells. The survival rate (usually measured by the proportion of BrdU+ neurons which have survived in the hippocampus for 28 days after BrdU incorporation) shows whether proliferating cells have survived for a period of time which is long enough to acquire a mature phenotype. Studies vary in the timing of cell survival assessed and therefore interstudy data comparisons should be done with caution. A differentiation assay with doublecortin (DCX) can show the amount of neuroblasts present in the hippocampus. This parameter represents the number of newly born neurons which have a potential to be incorporated into hippocampal networks, an important measure of the neuroplastic potential of the hippocampus owing to neurogenesis. Finally neural progenitor fate mapping shows the proportion of newly born cells which became neurons or astrocytes. It provides an insight into the neurogenic versus astrogenic balance of the differentiation in the dentate gyrus, which is thought to change depending on regulatory signals received by the hippocampus from the peripheral environment.

In summary, it is important to be cautious when comparing studies using different methodological approaches in the field of early-life effects on hippocampal neurogenesis, as even small variations in methods used can produce substantial differences between the models. However such variety also allows us to study the topic from different developmental and cellular points of view, which can ultimately provide a more comprehensive understanding of observed effects.

### 4.2. The Effect of Perinatal Immune Activation on Adult Hippocampal Neurogenesis: Research Conducted to Date

Studies investigating the early-life immune modulation of adult hippocampal neurogenesis available to date are summarised in Table 1. One of the first studies in the field was done by Meyer and colleagues [42]. In this study pregnant C57BL/6 mice were injected with poly I : C at GD 9 and GD 17 which, according to the authors, corresponded to the early-to-mid and mid-to-late periods of pregnancy. Immunohistochemistry using DCX marker showed a decrease in the number of young neurons in the dentate gyrus of prenatally exposed offspring at PND 24, which corresponds to the early adolescent period. This effect was independent of the timing of the prenatal treatment. However, behavioural effects observed in adulthood of the offspring showed a different picture. Behavioural tests done on the mice at the age of 14–16 weeks demonstrated that mice exposed to the maternal poly I : C response on GD 9 displayed increased anxiety-like behaviour in the open field test, while mice exposed to GD 17 expressed behaviours indicative of perseverations in the discrimination reversal learning task. These data are of interest as they suggest that immune activation at different stages of prenatal development can result in differential outcomes for behavioural abnormalities in adult life. It is interesting to speculate that behavioural differences in adulthood could be accompanied by the differences in neurogenesis which perhaps were not yet apparent in the adolescent period. Even though the measured parameters of neurogenesis in this study were limited, additional factors assessed in the hippocampus of these mice provided an insight into mechanisms which might cause the observed reduction in the number of young neurons. Indeed, DCX decrease was accompanied by the decrease in reelin, a protein which is known to regulate neurogenesis [46]. This evidence led the authors to suggest that the observed reduction could be due to increased apoptosis of progenitor cells. However, analysis of the apoptotic marker caspase-3 demonstrated that increased caspase-3-dependant apoptosis only took place in the hippocampi of GD 17 exposed animals, yet another indication of the time-dependant variation in the response to an immune challenge. The authors suggested that such variation was initially due to the differential foetal immune response to maternal inflammation. Specifically, they showed that while poly I : C injections resulted in an increase in cytokine levels in the brains of both GD 9 and GD 17 exposed foetuses, only GD 17 group had an accompanying increase in cytokine gene expression, suggesting that cytokine increase was due to endogenous production by the foetal brain in this group only.

A similar study design was later employed by Cui and colleagues [47]. This group also explored the consequences of maternal immune stimulation at different time points in pregnancy; however in their experimental design hippocampal neurogenesis was the primary focus of the study. More specifically, pregnant rats were peripherally administered LPS at a dose of 0.1 mg/kg on GD 15/16 (midgestation according to the study) or a dose of 0.05 mg/kg on GD 18/19 (late gestation). The dose difference was based on the increased sensitivity and mortality of near-term pregnant rats to LPS exposure. The results of BrdU incorporation assay showed that, by the end of the early perinatal period (the time between LPS injection and PND 14), the hippocampus of the GD 18/19 exposed offspring contained less cells which had undergone division, a decrease which might be due to decreased proliferation and/or survival of dividing cells. A similar trend was observed in the GD 15/16 group, but it did not reach significance. Interestingly, this situation was reversed when cell proliferation was assessed at PND 14: midgestation exposed animals had a significant decrease in hippocampal cell proliferation, while this trend did not reach significance in the late pregnancy exposed group. Furthermore, the number of cells which had undergone proliferation was significantly decreased in both conditions 4 weeks after BrdU incorporation, with the effect being more pronounced in the late gestation group. While this measure incorporates the effect of both cell proliferation and survival rates, this effect being stronger 4 weeks rather than 2 hours after BrdU incorporation suggests that indeed changes in cell survival rates might cause observed differences in the number of BrdU positive cells. Cell proliferation and survival in adult offspring (PND 60) were not affected; however the authors argued that even the deficit in proliferation and survival of neurons only in early development might have long term consequences, as some of these neurons could have been destined to survive and function for the lifetime of the individual. Finally, there was no change in neuronal differentiation in any of the described conditions.

These results were later challenged by another study which used prenatal LPS administration in pregnant rats. Graciarena and colleagues [44] employed a robust model of LPS administration which involved a higher dose of LPS (0.5 mg/kg) and repeated injections every other day between GD 14 and 20. Hippocampal neurogenesis was assessed only in adult offspring at a single time point of PND 60. Results showed that these animals had a consistent decrease in progenitor cell proliferation and neuronal differentiation into young and mature neurons arising from proliferating progenitors but the survival of proliferating cells was not affected. In addition, the authors also explored the role of microglia in the observed effects. They found that, in adult animals prenatally exposed to LPS, hippocampal microglia morphologically resembled stages II-III of activation. Interestingly the hippocampus was the only brain region where microglia displayed features of nonphagocytic activation, suggesting specific sensitivity of the hippocampus to LPS effects. The authors also explored cytokine expression in the hippocampus. At birth, prenatally exposed pups had increased IL-1*β* in the hippocampus (but not IL-6), as well as decreased anti-inflammatory cytokine transforming growth factor beta-1 (TGF*β*1). IL-1*β* levels were restored by adulthood consistent with the morphological appearance of microglia (nonphagocytic activation). However the levels of TGF*β*1 in the hippocampus remained reduced in adulthood of the exposed animals, which is of particular interest as TGF*β*1 has been shown to possess proneurogenic properties [48]. Also, overexpression of TGF*β*1 in the adult DG restored normal levels of neurogenesis in the LPS-exposed group, suggesting a causal role of TGF*β*1 in LPS-related decrease in hippocampal neurogenesis. Furthermore the observed effects had a behavioural correlate as LPS-exposed animals displayed impaired recognition memory in the novel object recognition test, while TGF*β*1 overexpression improved their performance in this memory task.

Moreover, in the follow-up study, the authors showed in an* in vitro* system that TGF*β*1 exerts its proneurogenic effect through the canonical Smad 2/3 pathway [49]. This study also highlighted a unique ability of prenatal LPS exposure to cause long-term neurogenic changes. While adult LPS exposure produced an acute reduction in cell proliferation similar in magnitude to prenatal LPS effect, it failed to affect cell differentiation in the long term as did prenatal exposure. Consistent with the hypothesis of TGF*β*1 involvement in these effects, TGF*β*1 levels were not affected by adult LPS exposure [49].

The studies described above provide an initial insight into the mechanisms underlying long-term changes in hippocampal neurogenesis and some behavioural correlates for these changes. However, in order to establish a relevant hypothesis of immune developmental programming, a more direct link with clinically-relevant psychopathology is needed. Introducing such a link, a recent study by Lin and colleagues [50] employed a similar prenatal exposure to an LPS paradigm to look at a depression-like behaviour in adulthood. In this study pregnant rats were injected with LPS at a low dose of 0.066 mg/kg on GD 10.5. As in the studies described previously, the prenatally exposed adult offspring displayed changes in hippocampal neurogenesis. Neurogenic changes were assessed at two time points: during the juvenile period at PND 21 and in adult rats (PND 90). The changes included a reduction in cell proliferation at both time points and a decreased number of DCX positive neuroblasts. Consistent with previous studies, there was no difference in the survival of proliferating cells or in the number of proliferating cells becoming mature neurons. Furthermore, the authors addressed whether these changes would predispose adult offspring to depression-like behaviour. The results of behavioural testing showed that, in adulthood, these rats more readily displayed learned helplessness in the forced swim test, a pharmacologically established model of a depression-like behaviour. Importantly, the exposed offspring showed a trend towards higher susceptibility to anhedonia measured by the levels of sucrose consumption in the course of the chronic mild stress paradigm, a widely used model to induce depressive-like behaviour in rodents by environmental stress. Strengthening the clinical link even further, the authors also showed that observed behavioural and neurogenic changes were rescued by a chronic administration of a common antidepressant fluoxetine. Thus prenatally exposed fluoxetine treated adult animals did not show increased learned helplessness behaviour in the forced swim test and their levels of cell proliferation and young hippocampal neurons returned to control level. However it is important to note that fluoxetine also increased neurogenesis in control animals, suggesting that fluoxetine might not necessary rescue LPS-damaged pathways but might be acting through an alternative mechanism. Nonetheless authors also showed that fluoxetine reversed some cellular abnormalities in the hippocampus which were suggested to underlie the mechanism of neurogenic changes, specifically brain derived neurotrophic factor (BDNF) expression in the hippocampus and dendritic spine density of the dentate granule neurons. These results suggest that hippocampal neurogenesis is indeed involved in the ability of perinatal immune activation to cause behavioural abnormalities in adulthood and increase adulthood susceptibility to stress-related psychiatric disorders such as depression.

It is important to note that all above-mentioned studies used a model of prenatal exposure, limiting the interpretation of the findings to prenatal conditions. However, a recent study by Järlestedt et al. [51] extended the early-life inflammatory exposure model to the postnatal period. In this study C57BL/6 mice were injected with a relatively high dose of 1 mg/kg of LPS at PND 9. Measurement of hippocampal neurogenesis found that while there was no acute effect on hippocampal cell proliferation in these pups at PND 11, in the juvenile period (PND 41), these animals already displayed a decrease in cell proliferation and in the number of progenitors becoming mature neurons and astrocytes. In adulthood (PND 60) these animals had a decreased number of cells which had undergone proliferation since the LPS injection (BrdU positive), a measure which is affected by both proliferation and survival of neural progenitors. Interestingly, this study was the first to show regional differences in the effect of LPS exposure, as the decrease in the number of BrdU positive cells was specific for dorsal hippocampus. These data are in line with the emerging evidence of the distinct functions of neurogenesis in different regions of the hippocampus [52].

Studies described so far, although different in many aspects of study design, provide converging evidence of the neurogenic consequences of perinatal immune activation. A common trend emerging from the data suggests that perinatally exposed adult animals tend to have a compromised hippocampal neurogenesis with a decrease in cell proliferation and neuronal differentiation being described more often than changes in cell survival.

Interestingly, Jiang and colleagues recently published two studies which can potentially provide an insight into what precedes the decrease in hippocampal neurogenesis in adult animals [53, 54]. Although they used a familiar model of prenatal exposure of rats to inflammatory stimulus at GD 15, their results should be compared with caution to the studies described previously as an exposure to a bacterial suspension of* E. coli* was used to stimulate immune response. This model, although potentially more relevant for reproducing clinical infection, is specific to the pathogen employed; therefore the data obtained from these studies cannot be directly extended to the effect of other environmental factors such as stress or nutrition on the immune system.

In these studies the state of hippocampal neurogenesis was evaluated during the first month of pup life at PND 3, 7, 14, and 28. Interestingly the authors showed that cell proliferation was increased during the first two weeks of life with a peak at PND 7 but returned to control levels by PND 28. The authors also describe some evidence for the increase in BDNF and its receptor expression and protein levels which coincide with a proliferation increase, a finding which supports the role of BDNF in the effect of immune stimulation on neurogenesis suggested by Lin and Wang [50]. Data from this study provide preliminary evidence that early-life immune activation can initially lead to a compensatory increase in hippocampal neurogenesis in early development followed by a subsequent decrease in the levels of cell proliferation and neuronal maturation in adulthood.

## 5. Conclusion

To summarise the findings described previously, perinatal inflammatory stimulation has a potential to modulate hippocampal neurogenesis in adult life. More specifically, animal studies available to date show that exposure to immune activation in the pre- or postnatal period reduces the rate of cell proliferation and neuronal differentiation in the adult hippocampus. Studies described in this review also suggest some cellular mechanisms which can underlie this long-term modulation, such as nonphagocytic microglial activation, a decrease in anti-inflammatory cytokines, and neurotrophic factors in the hippocampus. However further research is needed to uncover comprehensive pathways in an effort to develop interventions which could reverse such detrimental modulation.

Importantly, an inflammatory stimulus in early-life can result not only from infectious agents, but also from other environmental factors, such as stress and nutrition. Therefore the suggested hypothesis of developmental modulation of adult hippocampal neurogenesis by early-life immune activation can represent a converging common pathway through which various factors of early-life environment modulate hippocampal function in adult life. Such a pathway is of particular importance to psychiatric research as adult hippocampal neurogenesis has been implicated in many psychiatric diseases which are known to be exacerbated by early-life adverse events. Importantly, current research suggests a number of life style aspects which positively influence hippocampal neurogenesis, among them are exercises such as running and dietary factors such as polyphenols and omega-3 unsaturated fatty acids [55, 56]. If the hypothesis presented in this review is confirmed, this could suggest a beneficial effect of the therapeutic interventions affecting hippocampal neurogenesis for the promotion of mental health in individuals who experienced early-life adversities.

## Figures and Tables

**Table 1:** A summary of the studies investigating the effect of perinatal immune activation on postnatal hippocampal neurogenesis.

Reference	Animal model	Inflammation-stimulating agent	Time of exposure	Time of neurogenesis assessment	Effect on proliferation	Effect on survival	Effect on differentiation	Behavioural correlates	Evidence of underlying cellular mechanism
Cui et al., (2009) [47]	Pregnant Sprague-Dawley rats	LPS 0.1 mg/kg	GD 15/16	PND 14	Decrease	No significant effect	No effect	n/a	n/a
		LPS 0.05 mg/kg	GD 18/19	PND 14 PND 60 for both	n/a No effect	Decrease in prolif/survival^1 ^ No effect	No effect No effect		

Graciarena et al., (2010) [44]	Pregnant Wistar rats	LPS 0.5 mg/kg	GD 14–20 repeated injections every other day	PND 60	Decrease	No effect	Decrease in the number of cells becoming young and mature neurons	Impaired recognition memory in novel object recognition task	Microglial activation specific for DG; TGF*β*_1_ decrease in the DG

Graciarena et al., (2013) [49]	Pregnant Wistar rats	LPS 0.5 mg/kg	GD 14–20 repeated injections every other day	PND 60	Decrease in cell proliferation	No effect	Decrease in the number of cells becoming young and mature neurons	n/a	Microglial activationand TGF*β*_1_ and Smad2/3 pathway involvement
	Adult offspring	LPS 1 mg/kg	PND 60–68 injections every other day	PND 68	Decrease	No effect			Transient microglial activation, no change in TGF*β*_1_
				PND 120			No effect		
			

Järlestedt et al., (2013) [51]	C57BL6 mice	LPS 1 mg/kg	PND 9	PND 41	Decrease	n/a	Decrease in the number of neurons and astrocytes	n/a	n/a
				PND 60	n/a	Decrease in prolif/survival specific for dorsal hippocampus	n/a		

Jiang et al., (2012, 2013) [53, 54]	Pregnant Sprague-Dawley rats	*E. coli* (endocervical injection)	GD 15	PND 3, 7, 14, 28	Increase peaked at PND 7	n/a	n/a	n/a	Increase in BDNF and TrkB protein levels in the hippocampus
				PND 28	No change	No effect	No effect	Impaired spatial learning in MWM task	Normalised BDNF and TrkB protein content

Lin and wang (2014) [50]	Pregnant Sprague-Dawley rats	LPS 0.066 mg/kg	GD 10.5	PND 21 PND 90	Decrease Decrease	No effect No effect	Decrease in the number of young neurons Decrease in the number of young neurons	Increased expression of depressive-like behaviours	n/a

Meyer et al., (2006) [42]	Pregnant C57BL6 mice	Poly I:C 5 mg/kg	GD 9	PND 24	n/a	n/a	Increased number of young neurons in subgranular and outer granular layer	Increased adulthood anxiety in OF	Increased reelin in hippocampus
			GD 17	PND 24			Increased number of young neurons in outer granular layer of DG	Perseveration-like behaviour	Increased Caspase-3 in hippocampus

^1^Prolif/survival: a parameter measured by the total number of BrdU+ cells few weeks after BrdU incorporation which combines effects of proliferation and survival. OF: open field; DG: dentate gyrus.
